# What Does Current Literature Tell Us About the Mental Health of Looked After Children?

**DOI:** 10.1192/bjo.2022.200

**Published:** 2022-06-20

**Authors:** Katie Herwig

**Affiliations:** Mersey Care NHS Foundation Trust, Liverpool, United Kingdom

## Abstract

**Aims:**

Looked after children (LAC) are one of the most vulnerable groups in our society. Often, after experiences of neglect and abuse, they are more likely to experience poor mental health, attachment difficulties and problems in educational progress. This review aims to explore literature published over the past decade which addresses the mental health needs and management of LAC within CAMHS in the UK.

**Methods:**

A literature review was performed in compliance with the Preferred Reporting Items for Systematic Reviews and Meta-Analyses (PRISMA) 2009 structure. Selection criteria was used. The total number of papers identified after this initial search was thirty-six across the database.

Literature titles and abstracts were then screened to exclude papers with an irrelevant focus. Full-texts of the remaining twenty-two papers were then assessed for relevant and conclusive information. The total number of full papers included in the research was thirteen.

To analyse the literature identified, a framework of three themes has been highlighted. These include:

- the mental health needs of LAC,
- factors relevant to the assessment of LAC in mental health services
- considerations associated with the management of LAC with mental health conditions.

**Results:**

Mental health needs of LAC included numerous emotional, behavioural and social problems. These were largely focused around substance misuse, emotional disorders and poor relationships with peers.

The main themes which have come out of research in relation to the treatment of LAC include:

- the importance of supporting healthy social relationships with primary care-givers, peers and teachers
- maximising the informal support of family, friends and pets
- the provision of early, holistic and flexible mental health services, rather than disjointed agencies

**Conclusion:**

The literature published over the past decade has indicated the great number of adverse outcomes amongst LAC and has made useful suggestions for the assessment and treatment of these children within a CAMHS setting.

Through targeted support into residential placements, offering intensive and direct psychological input at an early stage and continuing even after they have been adopted, as well as, working alongside schools to promote peer interaction could significantly reduce the adverse outcomes of LAC.

Additionally, by referring patients along with their carers for psycho-education can be extremely beneficial. Alongside this, the young people ought to be directed to support groups with other LAC to meet peers who are in similar situations as themselves.

